# LiDAR-Based Negative Obstacle Detection for Unmanned Ground Vehicles in Orchards

**DOI:** 10.3390/s24247929

**Published:** 2024-12-11

**Authors:** Peng Xie, Hongcheng Wang, Yexian Huang, Qiang Gao, Zihao Bai, Linan Zhang, Yunxiang Ye

**Affiliations:** 1 School of Mechanical Engineering, Hangzhou Dianzi University, Hangzhou 310018, China; 222010111@hdu.edu.cn (P.X.); 222010126@hdu.edu.cn (Y.H.); 222010039@hdu.edu.cn (Q.G.); 212010101@hdu.edu.cn (Z.B.); zln@hdu.edu.cn (L.Z.); 2 Institute of Agricultural Equipment, Zhejiang Academy of Agricultural Sciences, Hangzhou 310012, China; yeyx@zaas.ac.cn; 3 Key Laboratory of Agricultural Equipment for Hilly and Mountainous Areas in Southeastern China (Co-Construction by Ministry and Province), Ministry of Agriculture and Rural Affairs, Hangzhou 310021, China

**Keywords:** negative obstacles, point cloud, orchards, LiDAR, tilted mount, unmanned ground vehicles

## Abstract

In orchard environments, negative obstacles such as ditches and potholes pose significant safety risks to robots working within them. This paper proposes a negative obstacle detection method based on LiDAR tilt mounting. With the LiDAR tilted at 40°, the blind spot is reduced from 3 m to 0.21 m, and the ground point cloud density is increased by an order of magnitude. Based on geometric features of laser point clouds (such as rear wall height and density, and spacing jump between points), a method for detecting negative obstacles is presented. This method establishes a mathematical model by analyzing changes in point cloud height, density, and point spacing, integrating features captured from multiple frames to enhance detection accuracy. Experiments demonstrate that this approach effectively detects negative obstacles in orchard environments, achieving a success rate of 92.7% in obstacle detection. The maximum detection distance reaches approximately 8.0 m, significantly mitigating threats posed to robots by negative obstacles in orchards. This research contributes valuable technological advancements for future orchard automation.

## 1. Introduction

In recent years, the use of mechanization in agricultural production has become increasingly popular in developing countries [1]. Agricultural robots need to perceive and understand the surrounding environment, with obstacle detection being crucial. Obstacles can be categorized based on their height relative to the ground into two types: positive obstacles, which are above the ground, and negative obstacles, which are below the ground [2]. Positive obstacles are easily detected by sensors as they are above the ground, whereas in orchard environments, there are numerous negative obstacles due to planting requirements. The negative obstacles mixed with the orchard roads make the information features not obvious enough, which pose a great risk to the orchard mobile robot. Negative obstacles, being below the ground, present a greater challenge for detection as only a small portion of their features can be detected. Some scholars have shown that the angle subtended by positive obstacles at the distance sensor R is proportional to 1/R, and the angle for negative obstacles is also proportional to $1/R^{2}$ [3], significantly increasing the difficulty of detection. Additionally, the complex orchard environment with vegetation cover further adds to the difficulty of detection [4]. Therefore, negative obstacle detection has always been a major challenge in the field of autonomous driving [3,5,6,7]. Negative obstacles in orchards change with planting needs, making the real-time detection of negative obstacles an urgent issue that needs to be addressed [8].

Over the past few decades, scholars have conducted extensive research on negative obstacle detection [9,10]. To ensure driving safety, the robot should detect both positive and negative obstacles as much as possible [11].The prevalence of negative obstacles in orchards poses significant safety hazards to robotic travel. As a result, efficiently detecting these obstacles in real time has become an urgent issue that needs to be addressed. There are currently three methods for detecting negative obstacles. The first is thermal imaging; the second is machine vision based on deep learning, and the third is detection methods based on LiDAR. Matthies et al. [3] proposed a negative obstacle detection method based on thermal infrared imaging, which mainly relies on the principle that negative obstacles are cooler in temperature than the surrounding area at night. By detecting areas with lower temperatures at night using the principles of thermal imaging, the spots of negative obstacles can be identified. However, this method only yields good detection results at night and is significantly affected by climate conditions.

Based on machine vision solutions utilizing depth cameras as the primary detection sensors, these approaches are mainly represented by deep learning algorithms [12,13,14,15]. Feng et al. [16] designed a segmentation network with dual semantic-feature complementary fusion to segment different negative obstacles on roads. Liu et al. [17] propose the YOLO-SCG model that enables faster and more accurate target detection. Koch et al. [18] compared the geometric features of potholes in asphalt pavement areas with flawless road surfaces to detect defective spots. Dhiman et al. [19] developed two stereo vision analysis methods and two deep learning methods for pothole detection. Machine vision-based methods are susceptible to environmental lighting conditions and may not function properly in nighttime environments. Silvister et al. [20] and Thiruppathiraj et al. [21] used convolutional neural networks on smartphones to detect pavement damage. Although the accuracy is high, the detection speed is relatively slow. Kang et al. [22] proposed an environmental perception algorithm that integrates a single-line LiDAR with vision to address the challenge of identifying small targets. However, the detection performance of this method is poor and cannot adapt to the complex environment of orchards.

LiDAR technology shows promising results, as it is less affected by adverse environmental conditions such as bad weather [23] or smoke [24]. Therefore, detection solutions based on LiDAR are currently more mainstream. LiDAR can accurately measure distances between obstacles and the sensor, with minimal impact from weather and lighting changes. Xiao et al. [25] proposed a point cloud enhancement technique. Larson et al. [26] identified potential negative obstacles by detecting gaps and an absence of data. Shang et al. [27] proposed a novel LiDAR setup method and introduced an algorithm based on feature fusion that employs Bayesian estimation for each weight, achieving promising experimental results; the new type of setup proposed in this paper gave us some inspiration, on the basis of which we have proposed our own installation. In the following text, we elaborate on the differences between our installation method and their installation methods. Park et al. [28] proposed a new 3D object detection method called temporal motion-aware 3D object detection (TM3DOD), which utilizes temporal LiDAR data; this method achieves comparable performance to state-of-the-art methods. This method is not very effective for the detection of small objects. Cheng et al. [29] proposed a negative obstacle feature detection method based on radial distance and local dense features with multiple LIDAR and synthetic features, which achieves better negative obstacle detection but poorer detection of small targets. Ravi et al. [30,31] proposed a LiDAR-based detection of cracks and potholes on highways and airport runways and estimation of negative obstacles area and volume; however, their study uses a 2D LiDAR that is prone to occlusions due to different types of road anomalies. Song et al. [32] propose a rule-based method for extracting target points from LiDAR ring data and edge triggers, which can improve detection speed and performance. The traditional method of installing LiDAR systems has the issue of large blind spots [27], which is highly detrimental to the detection of negative obstacles. This paper proposes a novel installation method for LiDAR to address this problem.

Zhou et al. [11] proposed a terrain perception technology that combines visual and LiDAR sensors for forest environments, estimating ground positions and extracting passable areas using visual sensors. Ruan et al. [33] developed a real-time negative obstacle detection model based on the YOLOv4 network for open-pit mine environments. Many scholars have also introduced technologies for detecting ground negative obstacles using unmanned aerial vehicle (UAV) platforms [34,35,36,37,38,39]. However, in orchard environments where vegetation cover obstructs visibility, drones struggle to detect terrain, rendering such methods ineffective in these environments. In addition, jiang et al. [40] investigated the detection of tree trunks in an orchard environment for the purpose of path planning and navigation.

The first section of this paper discusses relevant research on negative obstacle detection. The second section elaborates on the selection of LiDAR and proposes a novel installation method. On this basis, our paper presents a detection algorithm based on the spatial geometric features of negative obstacles and briefly introduces the experimental setup. The third section discusses the experimental results, conducting data analysis on the two experimental results regarding the installation method and the detection of negative obstacles, and concludes that the proposed method in this paper can meet the needs of re-al-time detection of negative obstacles in orchards. The fourth chapter summarizes the entire paper.

## 2. Methods

This paper proposes a detection algorithm that uses LiDAR to detect negative obstacles. To reduce the amount of blind spots and increase the ground point cloud density, an approach is adopted where the solid-state LiDAR is tilted during installation. Solid-state LiDAR is a new type of LiDAR that achieves a high point cloud density at a lower cost, which is highly beneficial for negative obstacle detection. Based on this, our paper detects negative obstacles using geometric features derived from point clouds. Since the LiDAR detection method is used, weather conditions, lighting, and temperature factors have less impact on the detection results, which can improve the robustness of the algorithm.

This section will provide a detailed introduction to the specific methods of negative obstacle recognition, mainly including the installation method of the LiDAR and the specific process of the negative obstacle recognition algorithm. Solid-state LiDAR is a type of three-dimensional LiDAR that has emerged in recent years, with advantages such as high reliability, compact structure, and lower cost, which has attracted the attention of scholars. This type of LiDAR is usually used for blind spot compensation. In this paper, a solid-state LiDAR is tilted and placed to scan the ground to obtain ground point cloud data, thereby achieving the detection of negative obstacles. On this basis, we establish a corresponding mathematical model based on the spatial geometric characteristics of the negative obstacle point cloud collected by the LiDAR and matched the characteristics of the negative obstacles with the mathematical model to achieve the detection of negative obstacles.

### 2.1. Tilted Installation of LiDAR

#### 2.1.1. The Disadvantages of Traditional Upright Setup

**Figure 1:**
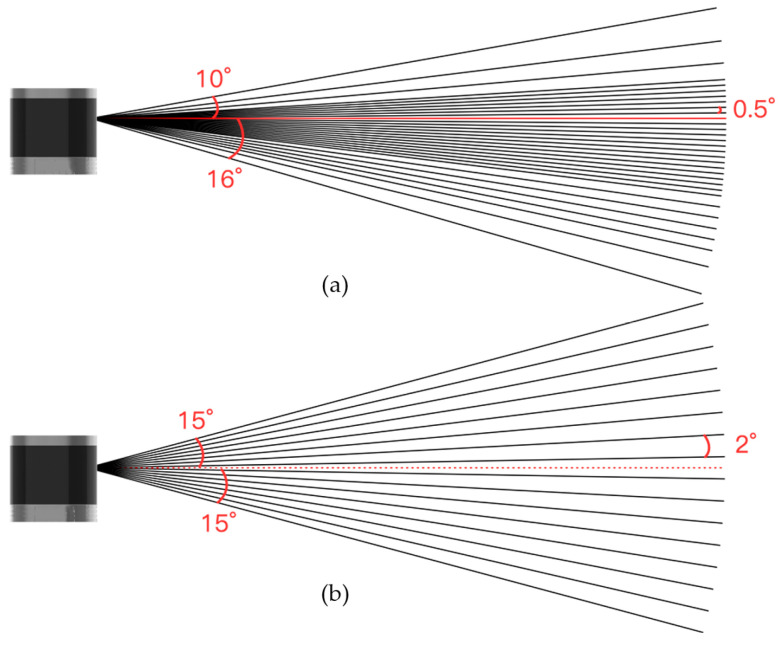
RoboSense LiDAR field of view (FOV) angle. Image from RoboSense official website: (**a**) Helios-32 LiDAR field of view angle and (**b**) Helios-16 LiDAR field of view angle.

The traditional upright setup of LiDAR for negative obstacle detection in orchards presents two significant disadvantages: (1) there is a large blind spot and (2) the point cloud density is relatively low. The traditional upright setup of LiDAR is typically designed for structured environments such as urban roads and indoor settings. In urban and indoor environments, negative obstacles are rare, so upright setup enables the LiDAR to scan further places. Within the blind spot, the risk to vehicles is minimal since negative obstacles are absent. However, this is not the case in orchard settings, where the presence of negative obstacles necessitates that the robot continuously detects whether there are negative obstacles in its vicinity (especially ahead) to avoid safety risks. As shown in Figure 1 (the figure is from the official website of RoboSense), which illustrates the beam distribution of a multi-line LiDAR (RoboSense, Shenzhen, China), it is evident that the vertical field of view (FOV) of the LiDAR in the image ranges from −15° to 15°.

**Figure 2:**
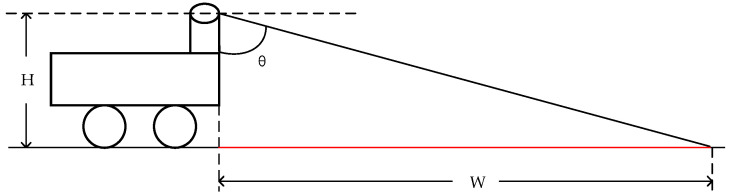
Diagram of blind spot in traditional upright LiDAR setup method.

As shown in Figure 2, it is a schematic diagram illustrating the blind spot for vertically installed LiDAR. Here, *H* represents the height from which the LiDAR emits the laser to the ground; *B* is the range of the blind spot, and *θ* is the angle between the lowest LiDAR beams and the vertical direction. It can be observed that

(1) W=H×tanθ.

Therefore, the extent of the blind spot for the traditional vertical setup method depends on the LiDAR height *H* and the angle *θ* between the lowest LiDAR beam and the vertical direction. Assuming the LiDAR installation height *H* is 0.8 m and the angle *θ* is 75°, we can calculate W=0.8×tan⁡75°≈3m. If the LiDAR is not installed in front of the vehicle but at the center of the vehicle, it may lead to a larger blind spot (part of the LiDAR beams might be obstructed by the vehicle’s edges). In complex environments like orchards, the occurrence of negative obstacles in the blind spot without real-time detection can be extremely dangerous.

**Figure 3:**
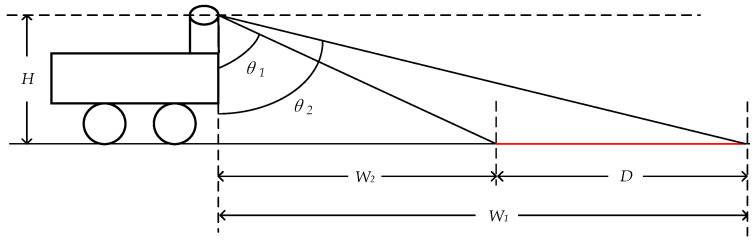
Diagram of wiring harness density in traditional upright LiDAR setup method.

The traditional vertical setup method also leads to the issue of too low a beam density on the ground. As shown in Figure 3 (for convenience of viewing, the angles depicted are different from the actual angles), it is a schematic diagram of the beam density of the traditional vertical LiDAR setup method. Here, D represents the distance between the two laser beams, and the angles between the two beams and the vertical direction are $\theta_{1}$ and $\theta_{2}$, respectively. $W_{1}$ and $W_{2}$ are the distances between the two laser beams and the front end of the vehicle, and H is the height from where the LiDAR emits laser to the ground. From this, we can derive

(2) $$D=W_{1}-W_{2}=H\times (\tan\theta_{2}-\tan\theta_{1}).$$

**Figure 4:**
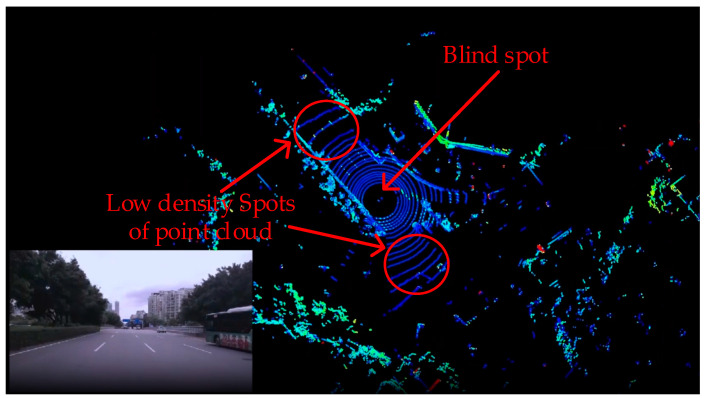
The disadvantages of traditional upright setup method in real environments.

Assuming H is 0.8 m, $\theta_{1}$ and $\theta_{2}$ are 75° and 73°, respectively. We can then calculate that D is 0.37 m. As $\theta_{1}$ and $\theta_{2}$ increase, the distance between the beams will further increase, leading to a low ground point cloud density, which makes it difficult to detect negative obstacles (as shown in Figure 3). Figure 4 illustrates two issues brought by the traditional vertical setup method: (1) a blind spot at close distances and (2) a low ground point cloud density at far distances.

#### 2.1.2. The Use of MID-70 LiDAR

In the negative obstacle detection method proposed in this paper, we use a MID-70 LiDAR (DJI, Shenzhen, China) instead of a mechanical rotating LiDAR. The MID-70 LiDAR is a type of solid-state LiDAR that has emerged in recent years. Compared to mechanical rotating LiDARs, solid-state LiDARs lack rotating mechanical parts, which reduce the failure rate and enhances durability. Moreover, these types of LiDARs are more affordable and have a compact structure, making them suitable for use in orchard environments. The traditional mechanical rotating LiDAR has a horizontal field of view of 360°, which allows it to scan the surrounding environment with a single rotation of the internal probe. However, this type of LiDAR is not suitable for negative obstacle detection in orchard environments due to two main reasons: (1) there is waste in the 360° field of view of the rotating LiDAR (more than half of the beams cannot illuminate the ground) and (2) the point cloud density of the rotating LiDAR is low (with point clouds distributed in every corner of space).

**Figure 5:**
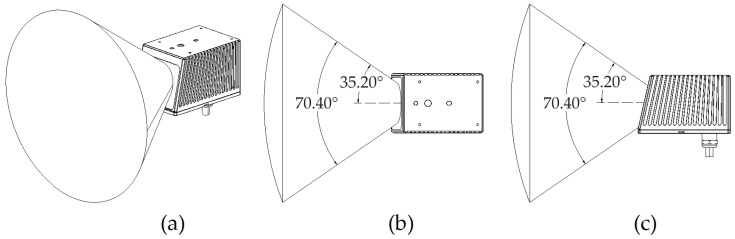
(**a**) Range of the LIVOX MID-70 LiDAR’s field of view (FOV); (**b**) horizontal field of view of the LIVOX MID-70 LiDAR; (**c**) vertical field of view of the LIVOX MID-70 LiDAR.

**Figure 6:**
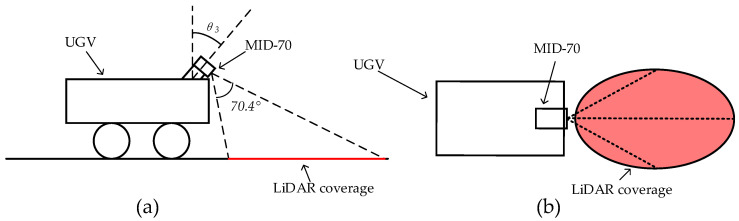
LiDAR setup method: (**a**) side view and (**b**) top view.

Addressing the above two issues, this paper utilizes the LIVOX MID-70 LiDAR for ground scanning as shown in Figure 5. This LiDAR has a circular field of view of up to 70.4° in both horizontal and vertical directions, with the near blind spot being decreased significantly to 21 cm. The larger field of view and smaller blind spot help the unmanned ground vehicle to detect the orchard’s environment more comprehensively and achieve real-time detection of negative obstacles. The inclined installation method allows most of the laser light to illuminate the ground (as shown in Figure 6). MID-70 adopts non-repetitive scanning technology, resulting in higher point cloud density, which enables accurate detection of every detail on the ground. Based on the aforementioned advantages, this paper proposes to use the MID-70 LiDAR as the sensor for detecting negative obstacles in orchards.

#### 2.1.3. The Tilted Installation of LiDAR

Considering the shortcomings of the traditional upright setup method, this paper proposes the solution of a tilted setup for LiDAR; compared with the traditional upright setup, the tilted setup allows more laser irradiation on the ground, as shown in Figure 6a. For the LiDAR setup schematic, the solid-state LiDAR is mounted in the center of the front of the vehicle and tilted at an angle $\theta_{3}$ in order to achieve the purpose of scanning on the ground. As shown in Figure 6b, the red area represents the scanning area of the LIDAR on the ground, in which $\theta_{3}$ can be adjusted to modify the scanning range of the LiDAR.

**Figure 7:**
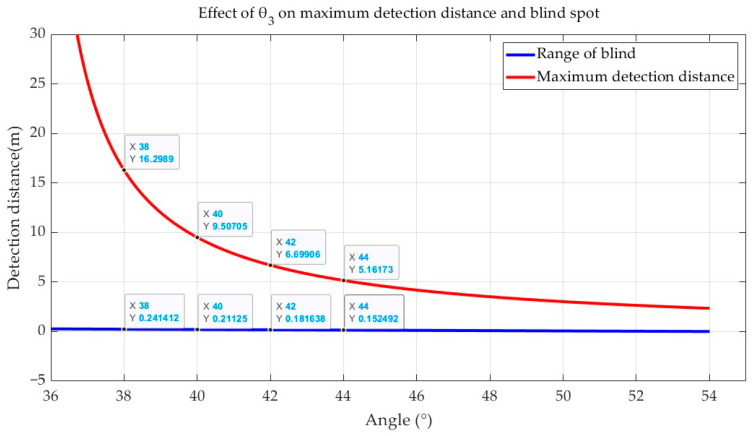
Effect of LiDAR tilt angle on blind spot and furthest detection range.

When the LiDAR is installed at a height of 0.8 m, the orientation of the LiDAR blind spot is $0.8\times \tan\left(54.8-\theta_{3}\right)$; the farthest detection distance is $0.8\times \tan\left(125.2-\theta_{3}\right)$, and the two functions are plotted in MATLAB as shown in Figure 7. The vertical viewing angle of the LiDAR is 70.4°, which is divided by the horizontal line, so theoretically, when $\theta_{3}$ is less than 35.2°, the farthest detection distance is infinity; when $\theta_{3}$ is greater than 54.8°, the blind spot will be reduced to 0, so Figure 7 plots the curve trend from 36° to 54°. After comprehensive consideration, this paper chooses a 40° tilt angle. In the case of the installation height of 0.8 m, the farthest detection distance is about 9 m, and the blind spot range is about 0.21 m; 40° is the angle at which we have experimented on, and we found the experiment to be more effective at this angle. When $\theta_{3}$ is smaller than 40°, although a longer detection distance can be obtained, the distant point cloud is too sparse (excessive distance between points will result in inconspicuous features of obstacles at a distance), resulting in detection effect that is not ideal; when $\theta_{3}$ is larger than 40°, although the blind spot range can be reduced, the detection distance will be greatly reduced, as can be seen from Equation (12). If the unmanned ground vehicle cannot detect the distant negative obstacles in time when traveling at a faster speed, this may lead to danger. In summary, a blind spot range of about 0.21 m and the farthest detection distance of about 9.5 m can meet the demand of negative obstacle detection in the orchard.

As can be seen from the blind spot curve plotted in Figure 7, under the tilted setup method, the LiDAR blind spot is significantly reduced, and almost all of the laser light from the MID-70 LiDAR can be irradiated on the forward ground area, greatly improving the density of the forward ground point cloud; this approach addresses the shortcomings of the traditional upright LiDAR setup. Under the tilt setup method, the geometric features of the negative obstacle point cloud are clearer than the traditional method, which enhance the robustness of the negative obstacle recognition system.

#### 2.1.4. Experimental Arrangements

We designed experiments to verify the advantages of the new mounting method proposed in this paper by placing four obstacles in front of the unmanned vehicle, scanning the obstacles with three different mounting methods, analyzing the number of point cloud on the obstacles and the range of blind spots, and comparing the advantages and disadvantages of the three mounting methods.

### 2.2. Negative Obstacle Detection Method Based on Point Cloud Spatial Geometric Features

Based on the inclined installation, we obtain the ground point cloud features, and by analyzing the spatial features of the negative obstacle point cloud, we can realize the detection of negative obstacles.

#### 2.2.1. Spatial Geometric Characterization of Negative Obstacle Point Cloud

Negative obstacle point clouds are characterized by the following three main features: (1) a jump in the spacing of neighboring points; (2) a “falling” phenomenon along at the wall after a negative obstacle point cloud; and (3) an increase in the density of the point cloud along the wall after a negative obstacle.

**Figure 8:**
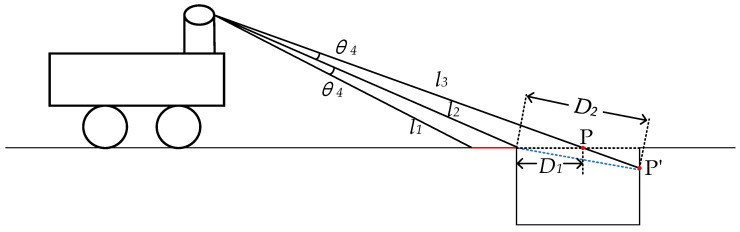
Laser point cloud distance jump at negative obstacles.

The geometric features of the negative obstacle are shown in Figure 8, and the angles between the three laser beams are all $\theta_{4}$, where laser $l_{1}$ is irradiated on the horizontal ground; laser $l_{2}$ is irradiated on the junction of the ground and the negative obstacle, and laser $l_{3}$ is irradiated inside the negative obstacle. When there is a negative obstacle, the irradiation points of $l_{3}$ changes from P to $P^{’}$, and the jump of the point cloud distance is a major geometric feature of the negative obstacle due to $D_{2}>D_{2}$.

**Figure 9:**
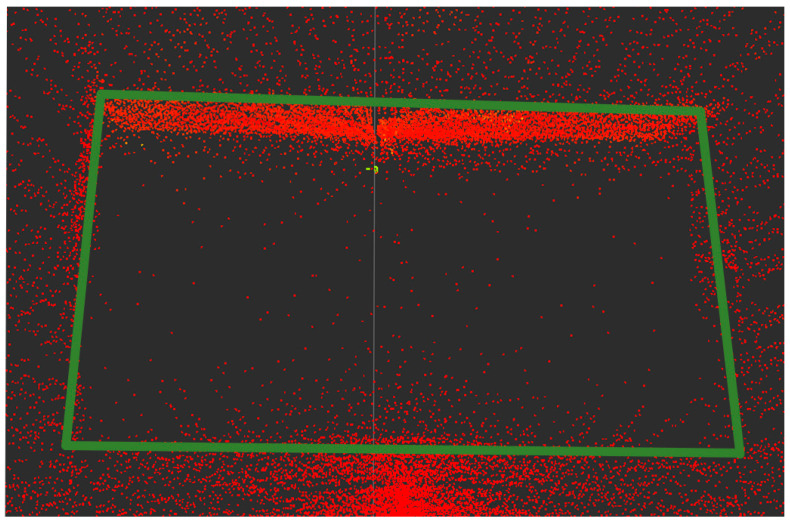
Negative obstacles point cloud.

Since the negative obstacle is lower than the horizontal ground, the point cloud inside the negative obstacle is also lower than the point cloud of the surrounding environment. Considering that the ground around the negative obstacle is not necessarily horizontal, this paper adopts straight-line fitting of the point cloud around the negative obstacle to represent the ground trend. The height of the point cloud at the negative obstacle is significantly lower than the fitted straight line, illustrating the characteristic of the point cloud “falling” along the wall after the obstacle. As shown in Figure 9, it is the actual point cloud diagram of the negative obstacle, with the green solid line, representing the boundary of the negative obstacle.

**Figure 10:**
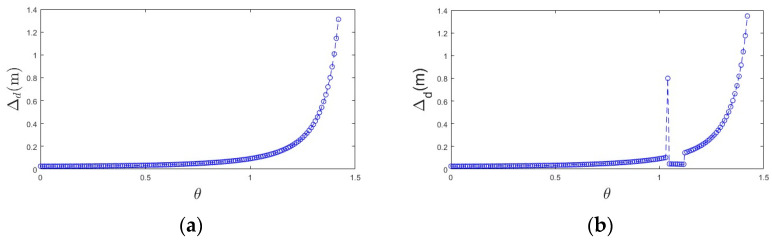
Relationship between point spacing and theta angle: (**a**) relationship between point spacing and theta angle in the absence of negative obstacles and (**b**) relationship between point spacing and theta angle in the presence of negative obstacles.

Negative obstacles also have the geometrical feature of dense point cloud along the back wall, as shown in Figure 10a; when there is no negative obstacle, the point cloud spacing (${∆}_{d}$) increases with the increase of θ angle, corresponding to the gradual decrease in point cloud density. However, when there is a negative obstacle, this gradual decreasing trend is interrupted. As illustrated in Figure 10b, the point spacing within the negative obstacle region is significantly reduced, leading to a marked increase in the density of points in this area.

#### 2.2.2. Negative Obstacle Detection: An Algorithm-Specific Process

**Figure 11:**
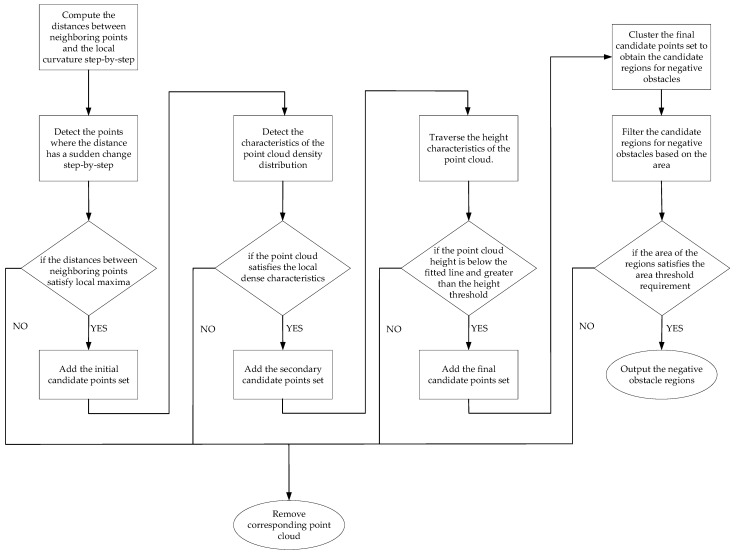
Flowchart of negative obstacle detection algorithm.

Through the above analysis, we learn that the negative obstacle point cloud has three different spatial point cloud distribution characteristics, based on which the negative obstacle can be detected. Each frame of the point cloud can be regarded as the signal of the LiDAR scanning the current ground environment, so as long as each frame of the point cloud is detected, the obstacle information can be extracted. Figure 11 shows the general flow of the negative obstacle detection algorithm in this paper.

In this paper, based on the spatial characteristics of the point cloud, we know that negative obstacles are characterized by proximal point jumps. Let the coordinates of a point be $P_{i}=[x_{i}\;,\;y_{i}\;,\;z_{i}]$, the point adjacent to $P_{i}$ and closer to the LIDAR is $P_{i-1}=[x_{i-1}\;,\;y_{i-1}\;,\;z_{i-1}]$. The neighboring point spacing of the point cloud can then be calculated as

(3) $$d_{n}^{i}=\sqrt{(x_{i}-x_{i-1}{)}^{2}+(y_{i}-y_{i-1}{)}^{2}+(z_{i}-z_{i-1}{)}^{2}}$$

We calculate the distance between neighboring points according to Equation (3) with a threshold ${Th}_{j}$. When the distance $d_{n}^{i}$ is greater than ${Th}_{j}$, the point involved in the calculation of $d_{n}^{i}$ is added to the initial candidate points set. In the experiments of this paper, we set ${Th}_{j}$ to 35 cm. By changing the value of ${Th}_{j}$ in different scenarios, it can adapt to the sizes of different negative obstacles. Based on the analysis presented earlier, it can be deduced that the abrupt change in the spacing of adjacent points marks the rear edge of a negative obstacle. Conversely, the laser point directly in front of this spacing jump corresponds to the front edge of the negative obstacle. The abrupt change in point spacing can roughly determine the location of the negative obstacle, reducing the subsequent computational load.

**Figure 12:**
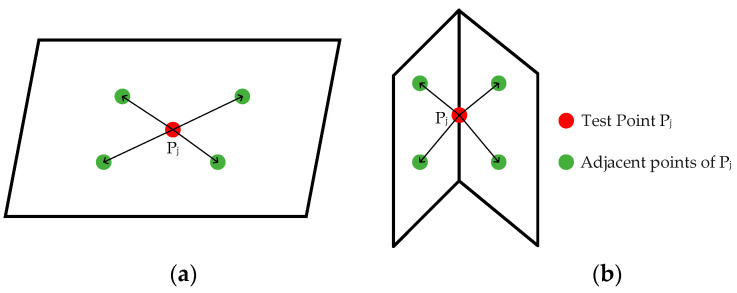
Local smoothness illustration: (**a**) $P_{j}$ is located on the surface and (**b**) $P_{j}$ is located on the line.

We define whether a point cloud has planar or linear features based on its smoothness; planar features indicate that the point is relatively smooth, while linear features suggest that the point is relatively steep. Point cloud smoothness can be calculated using Equation (4).

(4) $$S_{i}=\frac{1}{2n}∥\sum_{j\in [i-n,i+n],j\neq i}\frac{\left(P_{i}-P_{j}\right)}{∥P_{i}-P_{j}∥}∥$$

where *n* denotes the number of laser points selected to be neighboring *P_i_*; $S_{i}$ represents the magnitude of the sum of the unit vectors formed by point $P_{i}$ and its neighboring 2*n* laser points. As illustrated in Figure 12, when $P_{j}$ is situated on the surface, the individual unit vectors cancel each other out upon addition, resulting in a smaller $S_{i}$. Conversely, when $P_{j}$ is located on the line, the unit vectors do not cancel out upon addition, leading to a larger $S_{i}$. Take the face feature threshold ${Th}_{p}$ and the line feature threshold ${Th}_{l}$, when $S_{i}<{Th}_{p}$, the point is a face feature point, and when $S_{i}>{Th}_{l}$, the point is a line feature point.

After obtaining the initial candidate points set, we can then calculate the point cloud density. By filtering the *Z*-axis information of the point cloud, the point cloud is placed on a plane, which is divided into many small grids. The density of the point cloud is determined by the number of points in each grid. We set a threshold ${Th}_{s}$, and if the number of points in a grid exceeds ${Th}_{s}$, the corresponding three-dimensional point cloud points are added to the secondary candidate points set. If the condition is not met, the point cloud will be eliminated. We will compare the number of point clouds in the grid at the *m*-th row and n-th column with the number of point clouds in the surrounding grids, as shown in Equation (5).

(5) $$N_{p}\left(m,n\right)-N_{p}\left(i,j\right)>{Th}_{s},\;i\in [m-N_{g},m+N_{g}],\;j\in [n-N_{g},n+N_{g}]$$

where $N_{p}\left(m,n\right)$ represents the number of point clouds in the grid at the m-th row and *n*-th column, and $N_{g}$ denotes the range of the grid neighborhood.

We mentioned above that the negative obstacle point cloud has the characteristic of sinking along the wall. Afterward, we used a straight line fitted to the point cloud to represent the ground alignment and compare the height of the point cloud with the fitted straight line to obtain a collection of point clouds that are below the ground. Here, we describe the straight line with a point on the line and a vector in its direction. Let the space line pass through the coordinates $P_{0}=[x_{0},y_{0},z_{0}]$; this is a specific point on the straight line, which provides the exact position of the line in space, and the direction vector is n=[a,b,c]. This is the vector that defines the direction of the line. The components a,b,c of the direction vector n represent the unit lengths of the vector along the X, Y and Z axes, respectively, then the equation of the straight line is expressed as follows:

(6) $$\frac{x-x_{0}}{a}=\frac{y-y_{0}}{b}=\frac{z-z_{0}}{c}$$

**Figure 13:**
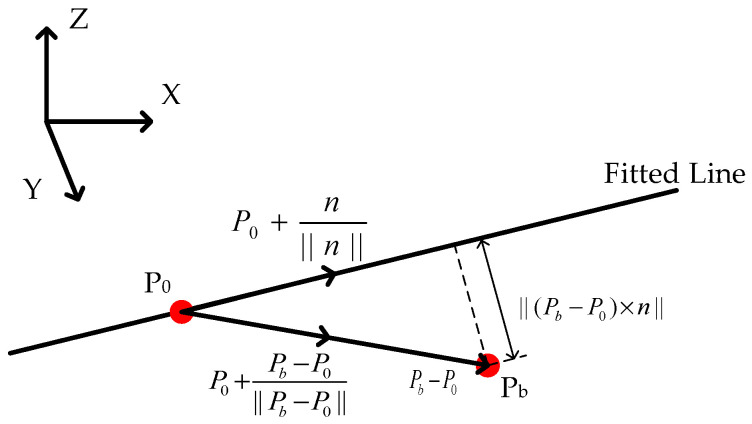
Fitting a straight line to the constraint relationship at points along the wall after a negative obstacle.

The distance between back-edge point $P_{b}$ and the straight line is

(7) $$||(P_{b}-P_{0})\times n||\geq {Th}_{d}$$

where *Th_d_* denotes the distance threshold. As shown in Figure 13, $P_{b}-P_{0}$ represents the vector from $P_{0}$ to $P_{b}$. By performing a cross product with n, the distance between $P_{b}$ and the fitted line can be calculated. Equation (7) indicates that the distance between point $P_{b}$ and the fitted line should be greater than or equal to ${Th}_{d}$. But since it is possible for the point cloud to be over a straight line, the following constraints are added:

(8) $$\left(P_{0}+\frac{P_{b}-P_{0}}{\left||P_{b}-P_{0}|\right|}\right)\cdot z<\left(P_{0}+\frac{n}{\left||n|\right|}\right)\cdot z$$

In Equation (8), where z is the unit vector in the direction of the *Z*-axis in the world coordinate system, as shown in Figure 13; $\frac{P_{b}-P_{0}}{\left||P_{b}-P_{0}|\right|}$ is the unit vector pointing from $P_{0}$ to $P_{b}$, and $\frac{n}{\left||n|\right|}$ is the unit vector along the fitted line. By adding $P_{0}$ to both vectors, we effectively translate the starting point of the vectors to $P_{0}$. Then, by taking the dot product with the z, we can calculate the magnitude of the components of both vectors along the *Z*-axis. This formula constrains the point cloud height to be below the fitted straight line and ensures that negative obstacles rather than positive obstacles are recognized. Under the constraints of Equations (7) and (8), points from the secondary candidate points set are added to the final candidate points set.

So far, we have obtained the final candidate point set for negative obstacles, but the negative obstacle size cannot be determined from these candidate point sets alone, and the potential obstacle size is obtained by point cloud clustering operation. Our principle is that the potential obstacle is allowed to be a little larger than the actual obstacle, but the potential obstacle is not allowed to be smaller than the size of the actual obstacle, otherwise it will possibly pose a safety hazard to the unmanned ground vehicle. In this paper, we use the mean-shift algorithm to cluster the candidate point cloud so as to calculate the polygon that describes the area of the negative obstacle. Finally, the negative obstacles are area filtered to filter out the negative obstacles with too small area and output the negative obstacle data.

**Figure 14:**
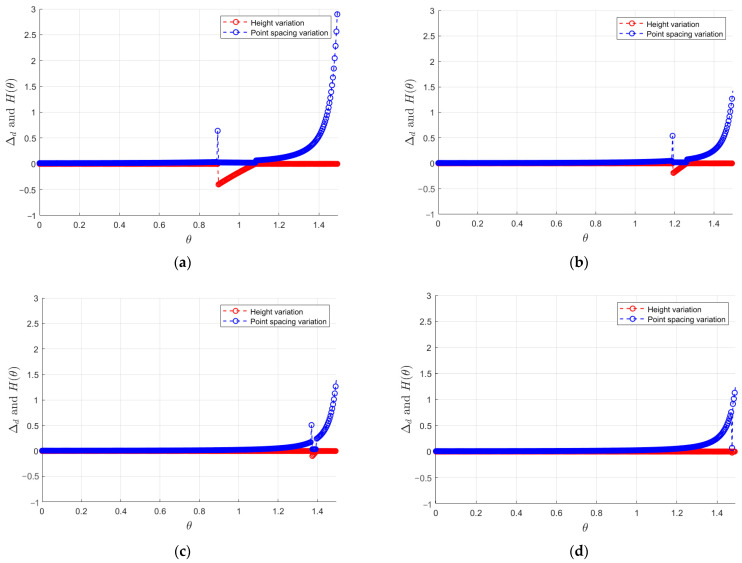
Point spacing and height of negative obstacles at different distances: (**a**) 1 m distance; (**b**) 2 m distance; (**c**) 4 m distance; and (**d**) 8 m distance.

In fact, the characteristics of negative obstacle point clouds vary at different distances. As shown in Figure 14, the point spacing and height features of obstacles are illustrated at distances of 1 m, 2 m, 4 m, and 8 m. It can be observed that when the negative obstacle is closer to the vehicle, the jump and height depression features of the point cloud are more pronounced. Conversely, when the distance is greater, the dense feature of the point cloud becomes more noticeable. Based on these characteristics, this paper sets different thresholds every 0.5 m to accommodate for the features of negative obstacles at varying distances.

#### 2.2.3. Experimental Arrangement

In this paper, experiments are designed to verify the feasibility of the algorithm in a real orchard; three types of negative obstacles are taken in the orchard and detected using the algorithm, respectively, and the detection results of each frame of point cloud are counted.

### 2.3. Experimental System and Evaluation Indicators

**Figure 15:**
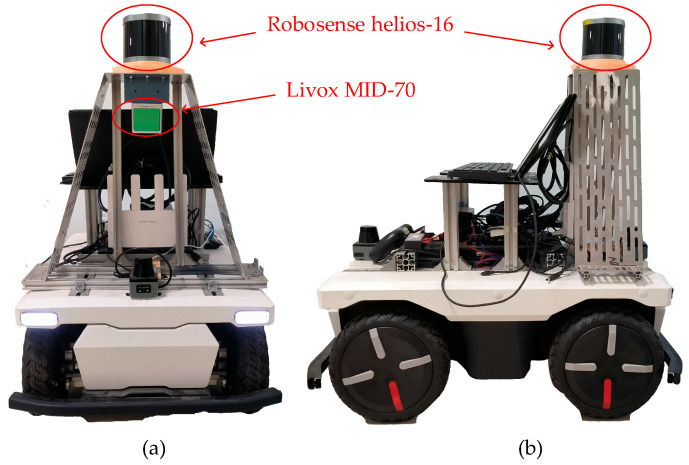
Experimental platform: (**a**) front view and (**b**) side view.

Figure 15 is the experimental system of this paper, which shows the main equipment used in the experiments, including a 16-line mechanical rotating LiDAR, a MID-70 solid-state LiDAR, and a cart platform (Yunle New Energy, Xuancheng, China).

In this paper, we separately count the detection results for different negative obstacles; we count the detection results of each frame and use these results as its performance-judgment index. The car travels towards the negative obstacle at a constant speed; when the distance between the vehicle and the obstacle is $D_{max}$, it starts to count the number of frames, and the total number of frames is $N_{0}$. In a detection process, there are four possible outcomes: the first is correctly identifying a negative obstacle, the second is identifying a negative obstacle while also marking a non-negative obstacle as negative, the third is only identifying non-negative obstacles, and the last is failing to detect any at all. Among these, the sum of the first and second scenarios is $N_{1}$; the sum of the second and third is $N_{2}$, and $N_{0}$ is the sum of all four situations. Based on these measurements, the detection success rate is defined as follows:

(9) $$P_{success}=\frac{N_{1}}{N_{0}}\times 100\%$$

False detection rate defined as follows:

(10) $$P_{false}=\frac{N_{2}}{N_{0}}\times 100\%$$

And miss detection rate defined as follows:

(11) $$P_{miss}=(1-\frac{N_{1}}{N_{0}})\times 100\%$$

We also count the negative obstacle time consumed per frame. When the negative obstacles are far away from the unmanned ground vehicle, the geometric features of the negative obstacles are not obvious, which lead to difficult detection, and too small negative obstacles are often filtered out as noise. In practical applications, the unmanned ground vehicle moves in the orchard at speeds of 5~20 km/h. According to Matthies et al. [41], it is known that the vehicle stopping distance can be determined using Equation (12).

(12) $$R=\frac{v^{2}}{2\mu g}+vT_{r}+B$$

where μ is the static friction coefficient between the ground and the wheels; the unmanned ground vehicle used in this paper is about 0.65 with the orchard ground; g is the gravitational acceleration, usually $g=9.8\;m/s^{2}$; $T_{r}$ is the total reaction time, usually 0.25 s; B is the safety buffer distance, usually 2 m in our experiments. When v>3.2 m/s(v>11.52 km/h), the speed value becomes the dominant term. When the speed is 20 km/h, the stopping distance can be calculated as 5.9 m using Equation (12). It can be seen that the detection distance of this algorithm meets the demand of the orchard scenario. The hardware device used in this paper is a computer with an Intel Core i5-1135G7 processor and 16 GB of RAM. The average computing time per frame is 17 ms, meeting the requirements for real-time detection.

## 3. Results

We designed three experiments to validate the advantages and disadvantages of the tilt-mounted LIDAR approach versus the traditional upright-mounted approach, as well as to validate the accuracy of the negative obstacle detection algorithm and the success rate of the detection in an orchard field scenario.

### 3.1. Experimental Result and Analysis of Tilt-Mounting Method

**Figure 16:**
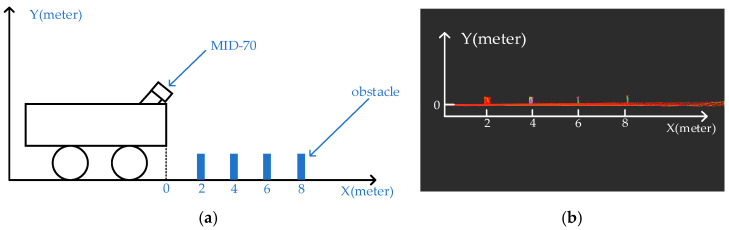
The visual range comparison experiments of the proposed setup: (**a**) schematic diagram of car and obstacles; (**b**) point clouds of obstacles.

To compare the differences among three installation methods, we designed three experiments: (1) tilted installation of solid-state LiDAR; (2) traditional upright installation; and (3) tilted installation of mechanical LiDAR. In the tilted installation experiment of solid-state LiDAR, this paper installed the MID-70 LiDAR at a 40° tilt on a platform 0.8 m above the ground; in the traditional upright installation experiment, the LiDAR was installed upright on a platform 0.8 m above the ground; in the tilted installation experiment of mechanical LiDAR, we installed the LiDAR at a 25° tilt on a platform 0.8 m above the ground. As shown in Figure 16, this paper placed four obstacles in front of the vehicle, each 21 cm high and 30 cm wide, with a 2 m interval between each obstacle, to verify the visible range of the LiDAR.

**Figure 17:**
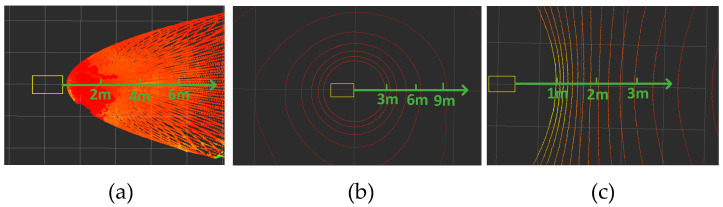
Ground point cloud with different mounting methods: (**a**) point cloud in inclined setup method; (**b**) point cloud in traditional upright setup method; and (**c**) point cloud in inclined mechanical LiDAR.

Figure 17a shows the field of view range of the MID-70 in the tilted mounting mode; Figure 17b shows the field of view range of the Helios-16 LiDAR in the conventional mounting mode; Figure 17c shows the field of view range of the Helios-16 LIDAR in tilt mount mode. The experimental study found that under the tilted mounting method, the LiDAR can scan more obstacles with a smaller field-of-view blind spot (see Table 1); while under the traditional upright setup method, the large field-of-view blind spot will not be able to detect the near obstacles, which will pose a safety hazard in a complex environment such as an orchard.

Another advantage of the proposed recommended mounting method is that the point cloud density is larger, which improves the LiDAR resolution and allows better analysis of negative obstacle spatial geometry features. As shown in Figure 17, we designed another experiment to verify this enhancement by placing an obstacle at 2 m, 4 m, 6 m, and 8 m away from the trolley, and all four obstacles are of the same size, labeled as S_1_, S_2_, S_3_ and S_4_, respectively. The obstacles are detected separately with two different setup methods of LiDAR, and then the number of point cloud on each obstacle is counted, because each obstacle has the same size, so the number of point clouds can directly respond to the density of point clouds, and the statistical results are shown in Table 1.

As can be seen from the data in Table 1, in the traditional upright setup, the number of point clouds on S_1_ is zero, because S_1_ is within the blind spot. On the other three obstacles, the number of point clouds was an order of magnitude higher in the tilt-mounted approach than in the traditional upright-mounted approach. Additionally, when we tilt the mechanical LiDAR for installation, the point cloud density increases, but it still does not match our proposed solution. Due to the mechanical LiDAR’s smaller vertical field of view, even with a tilt of only 25°, its maximum detection range is only 4.5 m, with a blind zone of 0.56 m. It can be concluded that by tilting the installation of solid-state LiDAR proposed in this paper, the number of point clouds on the obstacle is more, and the point clouds are denser, which are favorable for obstacle detection. Therefore, the tilted setup method proposed in this paper is advantageous for detecting negative obstacles in orchards.

### 3.2. Experimental Result and Analysis of Negative Obstacles Detection Algorithms

In order to evaluate the algorithm proposed in this paper, experiments were carried out in an orchard scenario. Our experiment consists of the following three parts: (1) introduction of the experimental site as well as the type and number of negative obstacles; (2) experimental evaluation metrics, mainly the detection of success rate and the maximum detection distance; and (3) the analysis of the reasons for false detection.

**Figure 18:**
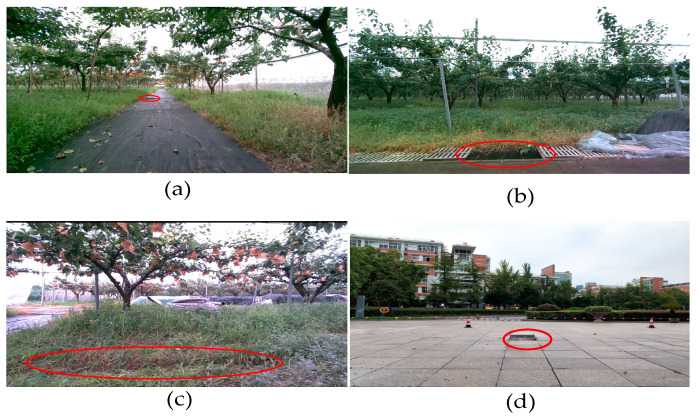
Three different negative obstacles in the orchard: (**a**) negative obstacle on the path to semi-structuring; (**b**) roadside sewer; (**c**) gully in the orchard; and (**d**) negative obstacle in the campus.

In order to verify the algorithm’s effect on the detection of negative obstacles, as shown in Figure 17. We setup negative obstacles in the orchard environment, and the orchard scene includes gullies and potholes, of which most of the gullies are manually excavated in the orchard mud, and the potholes include those obtained by digging downward in the mud and removing the sewer cover. Gullies are about 0.5 m wide and 0.5 m deep; potholes are 0.5–1 m in size and greater than 0.5 m deep. To show the generalization of the algorithm, we also added a negative barrier scene present in the campus, which is 0.6 m in size and about 0.3 m in depth. Potholes on semi-structured roads are shown in Figure 18a and are noted as negative obstacle O_1_; sewer openings alongside semi-structured roads are shown in Figure 18b and are noted as negative obstacle O_2_, and gullies alongside unstructured roads are shown in Figure 18c and are noted as negative obstacle O_3_; negative obstacles on campus are shown in Figure 18d and are noted as negative obstacle O_4_.

**Figure 19:**
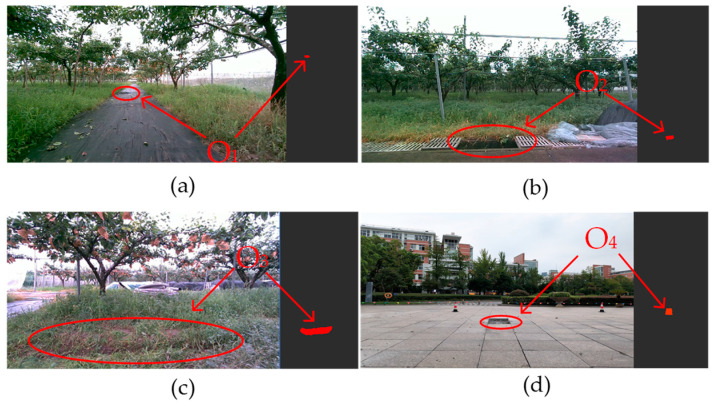
Detection results of the algorithm in three scenarios: (**a**) negative obstacle O_1_ test result; (**b**) negative obstacle O_2_ test result; (**c**) negative obstacle O_3_ test result; and (**d**) negative obstacle O_4_ test result.

In our experiments, we installed the MID-70 LiDAR tilted at 40° on a 0.8 m high vehicle platform and installed a camera below the LiDAR to capture images of the road ahead of the vehicle. Figure 19 shows the detection results for the different types of negative obstacles, with the camera capturing images on the left and the detection results on the right. The experiments show that the algorithm can effectively detect negative obstacles in the orchard.

In this paper, we first determined the statistical significance of maximum detection distance; we define the distance between the negative obstacle and the unmanned ground vehicle, when the negative obstacle is detected for the first time, as the maximum detection distance $D_{max}$; the maximum detection distance is one of the indexes used to measure the performance of the detection algorithm. In the orchard environment, the environment is more complex, and most of them are filled with semi-structured roads and unstructured roads. The road condition has a greater impact on the maximum detection distance; when the road is smoother and less shaded, the detection distance is farther; when the road is rugged and overgrown it is not favorable for negative obstacle detection. Therefore, for the same negative obstacle, the experiment is repeated to obtain the average value used for the final statistical results. In addition, the size of negative obstacles will also affect the maximum detection distance. Finally, the statistical results are listed in Table 2.

In this experiment, certain moments produced false alarms (*P_false_*) for negative obstacles; in the orchard, the environment is more complex due to various factors, such as rugged roads and overgrown weeds, so the point cloud shape is irregular and the likelihood of false alarms is much higher; the algorithm proposed in this paper is based on the detection of spatial geometrical features of negative obstacles, and therefore the probability of generating false alarms in unstructured environments is also higher. As shown in Table 2, the negative obstacle O_3_ in the unstructured scenario produces a higher false alarm rate than the other three scenarios.

## 4. Discussion

In this paper, we have installed the LiDAR at a height of 0.8 m on a platform with a 40° inclination angle, which we believe is an appropriate installation method. Based on the theoretical analysis presented earlier, it is known that increasing the height of the radar will increase the blind area and detection range, while increasing the inclination angle will decrease the blind area and detection range. In practical applications, the installation height and inclination angle can be adjusted appropriately to adapt to obstacle detection in different scenarios.

This paper proposes a method of tilt-mounting mechanical LiDAR to detect negative obstacles. The method of tilt-mounting mechanical LiDAR is also favored by some scholars. The article [27] also proposed the inclined installation of two mechanical LiDARs, that is, tilting two 32-line mechanical LIDARs in front of the vehicle. This method increases the point cloud density and narrows the range of blind spots. Our proposed inclined installation can also achieve this goal, but our solution has achieved better results at a lower cost. First, we have achieved a higher point cloud density. Table 1 shows that at a distance of 8 m from the vehicle, an obstacle with a surface area of 0.063 square meters has 161 point clouds. In the article by Shang et al. [27], the human body surface about 8 m away from the vehicle has 740 point clouds. If we convert the two to the same area (such as 1 square meter), our scheme has approximately 161 × (1/0.063) ≈ 2556 point clouds, while the article only has 740 × (1/0.935) ≈ 791 point clouds. The figure 0.935 represents half of the human body’s surface area, which was calculated using the formula provided in the article [42]. Therefore, the number of point clouds in our scheme is significantly better than that in the article’s scheme. Furthermore, although the blind spot range is not explicitly mentioned in the article [27], From the description in the article [27], it can be inferred that the blind spot area in front of the vehicle is approximately 0.5 m. which is larger than the 0.21 m blind spot in this paper. Finally, the cost of the LIDAR equipment we selected is much lower. As far as the author knows, the price of the HDL-32 LIDAR used in article [27] is about 52,000 US dollars, and the cost of the HDL-64 LIDAR is about 100,000 US dollars. The author of article [27] used two HDL-32 and one HDL-64 LIDARs, with a cost exceeding 200,000 US dollars. The cost of the MID-70 LIDAR used in this article is around 1000 US dollars.

It is easy to find from the above experiments that the farthest detection distances of negative obstacle environments are O_2_ and O_4_ with relatively flat roads is farther. This is because the structured environment is free from the influence of weeds and rough roads, which is beneficial for negative obstacle detection. At the same time, unstructured scenes will also increase the number of false alarms. This is because the point cloud shape of unstructured scenes is irregular, affecting the extraction of spatial features of ground point clouds, leading to an increase in the false alarms rate and a decrease in the successful detection rate.

This paper uses three thresholds to filter the three characteristics of negative obstacles. In our experimental process, we found that the stricter the threshold selection, the lower the success rate of negative obstacle detection, while the false alarm rate also decreases accordingly. Conversely, the more lenient the threshold selection, the higher the success rate, but the false alarm rate also increases.

Despite these difficulties, this paper still chose to compare with some of the more advanced negative obstacle detection algorithms currently available. Dhiman et al. [19] proposed a stereo vision-based detection scheme that can detect potholes whether they are dry, water-filled, or snow-covered; the overall accuracy of the 50 datasets presented in the paper is 88%, slightly lower than the method proposed in this paper. The paper does not mention the maximum detection distance, so a comparison cannot be made; however, to the authors’ knowledge, the detection distance accuracy of vision-based detection scheme is usually lower than that of LiDAR detection.

Unfortunately, to the best of the authors’ knowledge, there is currently no public dataset available for negative obstacle detection. Due to the varying sizes of negative obstacles and their prevalence in non-structured environments, it is challenging to define a negative obstacle scenario. Moreover, the detection scenario can also affect the success rate and detection distance. Orchard environments are particularly complex, as it is difficult to receive GNSS signals due to tree cover, and the obstruction of light by trees can also affect the operation of machine vision. Furthermore, due to the presence of ground weeds, it is almost impossible for LiDAR to detect the ground beyond 10 m. Therefore, it is difficult to compare negative obstacle detection in orchard environments with other environments. In the real world, the number of negative obstacles is much less than that of positive obstacles, making the collection of datasets relatively difficult and resulting in insufficient data volume. The next step should be to further collect more negative obstacles to improve the robustness of the algorithm. The experiments conducted in our paper were carried out in a real orchard environment. Due to the actual planting requirements, the spacing between negative obstacles is relatively large, which makes it difficult to find two negative obstacles within a limited detection range. Therefore, there is indeed a challenge in conducting experiments for the simultaneous detection of multiple negative obstacles. In future research, we plan to conduct experiments on multi-obstacle detection and discuss possible implementation strategies.

## 5. Conclusions

In this paper, a detection algorithm based on LiDAR for detecting negative obstacles in orchards is introduced. The paper proposes both a method for tilt-mounted LiDAR and a detection technique based on the geometric features of negative obstacle point clouds. As a purely laser-based detection method, it ensures reliability and robustness in the complex environment of orchards. The work presented in this paper focuses on two main aspects: (1) proposing the tilt-mounted LiDAR method and (2) developing a detection algorithm based on the spatial geometric features of negative obstacle point clouds using the tilt-mounted LiDAR.

The MID-70 solid-state LiDAR chosen in this paper is an economical choice. Solid-state LiDAR has a lower cost but offers a higher field of view, which is very favorable for negative obstacle detection. Under the tilt-mounting method, the density of the ground point cloud in front of the unmanned ground vehicle increases dramatically; the point cloud density is increased by more than an order of magnitude, which greatly improves the resolution of the ground point cloud; compared with the traditional upright-mounting method, the tilt-mounting LiDAR method can reduce the blind area range from 3 m to 0.21 m, which ensures the safety of the vehicle. In conclusion, the tilt-mounted MID-70 solid-state LiDAR can ensure that the majority of the laser beams are illuminated on the ground, which improve the robustness of the negative obstacle detection system.

On this basis, the geometric characterizations of the negative obstacle point cloud are used to analyze the difference between the negative obstacle and the horizontal ground, and the three features of the negative obstacles in space are analyzed to detect point pairs on the laser-scanning line that correspond to negative obstacles, and the candidate point sets are clustered and filtered to finally obtain the negative obstacle detection results. The detection success rate in the orchard field scene reaches 92.7%, and the average time consumed per frame is about 17.3 ms, which meets the real-time detection and obstacle avoidance requirements of the orchard.

The experimental results show that this method can realize the demand for real-time detection of negative obstacles in the orchard and improve the obstacle avoidance ability of orchard robots. The purpose of this paper is to improve the robot’s ability to detect negative obstacles in the orchard and to address the rollover and tipping issues caused by negative obstacles in the orchard.

## Figures and Tables

**Table 1:** The points distribution on obstacles under the tilted setup and the traditional setup.

Setup Method	S_1_	S_2_	S_3_	S_4_
Traditional upright mount	0	36	22	18
The tilt mechanical LiDAR	105	82	27	25
The tilt solid-state mount	835	551	264	161

**Table 2:** Statistics of negative obstacle detection result.

Negative Obstacle Scenario	*N*_0_	*N*_1_	*N*_2_	*P_success_*	*P_false_*	*P_miss_*	*D_max_* (m)	Average Time Spent (ms)
O_1_	525	490	30	93.3%	5.7%	6.7%	8.0	16.2
O_2_	684	635	36	92.8%	5.3%	7.2%	8.5	17.1
O_3_	396	352	32	88.9%	8.1%	11.1%	6.9	18.9
O_4_	384	368	10	95.8%	2.6%	4.2%	8.4	16.8
average	497	461	27	92.7%	5.4%	7.3%	8.0	17.3

## Data Availability

Data are contained within the article.
